# Association between the functional polymorphism Ile31Phe in the *AURKA* gene and susceptibility of hepatocellular carcinoma in chronic hepatitis B virus carriers

**DOI:** 10.18632/oncotarget.18613

**Published:** 2017-06-27

**Authors:** Zhiyu Bao, Lei Lu, Xinyi Liu, Bingqian Guo, Yun Zhai, Yuanfeng Li, Yahui Wang, Bobo Xie, Qian Ren, Pengbo Cao, Yuqing Han, Weihua Jia, Minshan Chen, Xinqiang Liang, Xuan Wang, Yi-Xin Zeng, Fuchu He, Hongxing Zhang, Ying Cui, Gangqiao Zhou

**Affiliations:** ^1^ State Key Laboratory of Proteomics, Beijing Proteome Research Center, Beijing Institute of Radiation Medicine, Beijing, China; ^2^ Guangxi Medical University, Nanning, China; ^3^ Affiliated Hospital of Jining Medical University, Jining, China; ^4^ National Engineering Research Center for Protein Drugs, Beijing, China; ^5^ National Center for Protein Sciences Beijing, Beijing, China; ^6^ Department of Surgical Oncology, Bayi Hospital Affiliated Nanjing University of Chinese Medicine, Jindu Hospital, Nanjing, China; ^7^ State Key Laboratory of Oncology in Southern China, Guangzhou, China; ^8^ Department of Experimental Research, Sun Yat-Sen University Cancer Center, Guangzhou, China; ^9^ Department of Hepatobiliary Oncology, Sun Yat-Sen University Cancer Center, Guangzhou, China; ^10^ Anhui Medical University, Hefei, Anhui, China

**Keywords:** hepatocellular carcinoma, hepatitis B virus, *AURKA*, polymorphism, susceptibility

## Abstract

Aurora kinase A (AURKA) is a serine threonine kinase which affects chromosomal separation and mitotic spindle stability through interaction with the centrosome during mitosis. Two functional nonsynonymous polymorphisms of the *AURKA* gene (Ile31Phe and Val57Ile) have been reported recently. We analyzed the association between the two polymorphisms and risk of the occurrence of hepatitis B virus (HBV)-related hepatocellular carcinoma (HCC) in the Guangxi population consisting of 348 patients with HCC and 359 control subjects, and then validated the significant association in the Guangdong population consisting of 440 cases and 456 controls. All of the participants were of Chinese origin and HBV carriers. The two polymorphisms were genotyped by polymerase chain reaction-restriction fragment length polymorphism assay or Sequenom MassARRAY iPLEX platform. In the Guangxi population, carriers of the *AURKA* 31Phe allele (Ile/Phe + Phe/Phe) were significantly associated with decreased susceptibility to HBV-related HCC when compared with noncarriers (Ile/Ile) (odds ratio [OR] = 0.63, 95% confidence interval [CI] = 0.46-0.86, *P* = 3.4 × 10^-3^). On the contrary, no significant association was found between Val57Ile and HBV-related HCC occurrence. The association of Ile31Phe with HBV-related HCC occurrence was confirmed in the Guangdong population (OR = 0.64, 95% CI = 0.49-0.83, *P* = 8.0 × 10^-4^). The pooled analysis gave a joint *P* value of 5.5 × 10^-6^ (joint OR = 0.63, 95% CI = 0.52-0.77). Our findings suggest that *AURKA* Ile31Phe may play a role in mediating the susceptibility to HBV-related HCC among Chinese.

## INTRODUCTION

Hepatocellular carcinoma (HCC) is the fifth most common cancer in the world and ranks the third as a cancer killer due to its high fatality rate [1]. It has been reported that about half a million new cases of HCC emerged in China each year [2]. It is well known that multiple risk factors contribute to hepatocarcinogenesis, such as chronic hepatitis B virus (HBV) infection, excessive alcohol consumption, cirrhosis, carcinogen exposure (such as aflatoxin B1), and a number of genetic and epigenetic alterations [3, 4]. Though HBV infection is the major cause of HCC, only a fraction of infected patients develops HCC during their lifetime, suggesting that genetic factor may play an important role in individuals’ susceptibility to HCC.

Genetic instability, which can generate mutations in oncogenes and tumor-suppressor genes, is a main driving force for malignant transformation and tumor progression [5, 6]. The centrosomes can play an extremely important role in the maintenance of genomic stability by organizing bipolar spindle during mitosis and by ensuring equal segregation of replicated chromosomes into daughter cells [7]. Aurora kinase A (AURKA, also known as STK15, BTAK and AIKI), which is implicated in the regulation of the G_2_/M transition and acts as a key regulator in chromosome segregation and centrosome functions [8, 9], localizes to centrosomes and mitotic spindles where it mediates mitotic progression and chromosomal stability. Previous studies have shown that amplification or overexpression of *AURKA* leads to centrosome amplification, bypass of the G_2_/M DNA-damage checkpoint, chromosome instability, malignant transformation, and tumor progression, suggesting that *AURKA* may play an important role in these oncogenic processes [10–19]. In accordance with these findings, amplification, overexpression, and/or constitutive activation of AURKA have been reported in many types of human cancers and cancer cell lines [20–23].

Recently, *Aurka* has been identified as a new direct transcriptional target of p53 in mouse liver, and the direct repression of *Aurka* by p53 in quiescent liver may be necessary to suppress the tumor-promoting consequences of the overexpression of AURKA in liver [24, 25]. In another study, the tumor suppressor SIRT2 has been found to interact with and degrade AURKA, partially accounting for the observation that *Sirt2*-deficient male mice would develop more HCC than wild-type mice [26]. In addition, Dauch *et al*. have found that in the context of chronic liver damage in mice, AURKA can bind to and stabilize phosphorylated MYC, overcoming G_2_/M cell-cycle arrest and promoting survival of liver cancer cells [27]. In accordance with its role as an oncogene, AURKA is overexpressed at mRNA and protein levels in HCC cell lines and in more than 60% HCC patients [28, 29]. Moreover, the high level of *AURKA* mRNA expression in HCC is associated with high-stages and poor outcome [28, 29].

The *AURKA* gene is located on chromosome 20q13.2. Recently, a nonsynonymous polymorphism Ile31Phe (F31I, rs2273535) has been identified in the exon 4 of *AURKA*, which locates at nucleotide position 91 and encodes a phenylalanine (Phe)-to-isoleucine (Ile) substitution at amino acid position 31. Ile31Phe is a structurally nonconservative aromatic/aliphatic amino acid substitution, and exists in an evolutionarily conversed region of the NH2-terminal domain which may function in translocation of the AURKA protein from cytoplasm to centrosome during mitosis [11, 30]. Functional analyses have revealed that the 31Ile variant is preferentially amplified and associated with aneuploidy in human colon tumors, and more readily enhanced both cell growth *in vitro* and tumorigenicity in nude mice compared with the 31Phe counterpart [31]. Although AURKA 31Phe was expressed at a level similar to that of AURKA 31Ile, the 31Phe variant binds more preferentially than 31Ile to the E2-ubiquitin-conjugating enzyme UBE2Nin human cells and is colocalized with UBE2N in the centrosomes, and this interaction correlates with attenuation of the ability of AURKA to induce cell growth and transformation [31]. In addition to Ile31Phe, another nonsynonymous polymorphism rs1047972 has been identified in the exon 4 of *AURKA*, resulting in valine/isoleucine at amino acid 57 (Val57Ile, V57I). The different haplotypes constituted by Ile31Phe and Val57Ile show variable functional consequences [32]. First, kinase activity levels of 31Ile-57Ile and 31Phe-57Ile were reduced to 15% and 40% compared with 31Ile31-57Val *in vivo* and *in vitro*. Second, abnormal nuclear morphology was 30 to 40 times more frequent in human immortalized fibroblast cells overexpressing 31Ile-57Ile or 31Phe-57Ile compared with the other two haplotypes. Finally, genomic instability of esophageal squamous cell carcinomas (ESCs) in the 31Phe-57Ile homozygotes was about 10-fold higher than that in the 31Ile-57Val homozygotes. Having these findings in mind, we expect that the two functional polymorphisms (Ile31Phe and Val57Ile) in the *AURKA* gene may act as genetic modifiers in individual susceptibility to human cancer. Indeed, several epidemiological studies have investigated the association between the two polymorphisms and the risk of breast, colon, esophageal, uterine, ovarian, prostate, skin and lung cancer, although the results have not been consistent [33–42]. However, according to our best knowledge, no studies have been conducted to specifically evaluate the two polymorphisms and risk of HCC. Therefore, in this study we performed association study in two case-control populations of Chinese ancestry, to examine whether functional polymorphisms in the *AURKA* gene has any bearing on the occurrence of HBV-related HCC among Chinese.

## RESULTS

### Summary description of the subjects participated in this study

The selected characteristics of the two case-control populations were summarized in Supplementary Table 1. In the Guangxi population, as described previously [43–45], there was no significant difference between patients and control subjects in terms of sex distribution, smoking and drinking status, and pack-years of smoking (all *P* values > 0.05). However, higher mean age (*P* = 1.2 × 10^-6^, *t* test), more subjects with age greater than 45 years (*χ^2^* = 11.1, *P* = 9.6 × 10^-4^), and more subjects with first-degree family history of HCC (*χ^2^* = 19.1, *P* = 1.2 × 10^-5^) were presented in the cases compared with controls (Supplementary Table 1). As previously reported, the Guangdong case-control population contained 751 cases and 509 controls [43]. However, 311 cases and 53 controls were excluded in this study because their DNA had been depleted in the original study. Therefore, the present Guangdong population had a total of 440 cases and 456 controls. No significant differences were observed in terms of the age and sex distribution between the previous 751 and the present 440 cases (mean age, 49.3 and 49.1 years, respectively; male/female ratio, 6.6 and 6.1, respectively), and between the previous 509 and the present 456 controls (mean age, 48.1 and 47.8 years, respectively; male/female ratio, 5.3 and 4.8, respectively) (Supplementary Table 1) [43]. In the Guangdong population, cases and controls were comparable with regard to mean age, and age and sex distributions (all *P* values > 0.05). The data on smoking and drinking status, pack-years of smoking, and family history of HCC were largely not available in this case-control population (Supplementary Table 1).

### Genetic association between *AURKA* Ile31Phe and HBV-related HCC among Chinese

The genotyping results of Ile31Phe and Val57Ile in the Guangxi population are shown in Table 1. The observed genotype frequencies for the two polymorphisms conformed to the Hardy-Weinberg equilibrium (HWE) in both cases and controls (all unadjusted *P* values > 0.05). For Ile31Phe, the frequencies of the Ile/Ile, Ile/Phe and Phe/Phe genotype among cases were significantly different from those among controls (*χ*^2^ = 8.4, *P* = 0.015, *df* = 2), and this difference was mainly caused by a higher frequency of the Ile/Ile genotype among cases compared with controls (53.7% vs. 42.9%). On the basis of logistic regression analysis with adjustment for age, sex, status of smoking and drinking, pack-years of smoking, and family history, subjects bearing Phe allele (Ile/Phe + Phe/Phe) had a significantly decreased susceptibility to HBV-related HCC compared with those bearing Ile/Ile genotype (OR, 0.63; 95% CI, 0.46-0.86; *P* = 3.4 × 10^-3^). The association withstood Bonferroni correction for multiple comparisons. For Val57Ile, we found no significant association with the risk of HCC (OR, 0.79; 95% CI, 0.56-1.12; *P* = 0.18). We also performed haplotype analyses of these two SNPs (in the order of Ile31Phe and Val57Ile; Supplementary Table 2). As compared with the haplotype Ile-Val, the haplotype Phe-Val was significantly associated with HCC risk (unadjusted *P* value = 0.016). However, the haplotype was much inferior to Ile31Phe in terms of the significance of the association, suggesting the association is only attributed to Ile31Phe.

**Table 1:** Association results for *AURKA* polymorphisms in two case-control populations

Polymorphisms	Genotypes	Cases, n (%)	Controls, n (%)	OR (95% CI)	*P* value
Ile31Phe					
Guangxi population		n = 348	n = 359		
	Ile/Ile	187 (53.7)	154 (42.9)	1.00 (Reference)	
	Ile/Phe	131 (37.6)	164 (45.7)	0.62(0.45-0.87)	4.9 × 10^-3^
	Phe/Phe	30 (8.6)	41 (11.4)	0.66(0.38-1.12)	0.12
	Ile/Phe + Phe/Phe	161 (46.3)	205 (57.1)	0.63 (0.46-0.86)	3.4× 10^-3^
Guangdong population		n = 440	n = 456		
	Ile/Ile	234 (53.2)	193 (42.3)	1.00 (Reference)	
	Ile/Phe	164 (37.3)	214 (46.9)	0.62 (0.47-0.82)	8.5 × 10^-4^
	Phe/Phe	42 (9.6)	49 (10.8)	0.71 (0.45-1.13)	0.15
	Ile/Phe + Phe/Phe	206 (46.8)	263 (57.7)	0.64 (0.49-0.83)	8.0 × 10^-4^
Pooled population		n = 788	n = 815		
	Ile/Ile	421 (53.4)	347 (42.6)	1.00 (Reference)	
	Ile/Phe	295 (37.4)	378 (46.4)	0.62 (0.50-0.77)	9.3 × 10^-6^
	Phe/Phe	72 (9.1)	90 (11.0)	0.67 (0.47-0.94)	0.022
	Ile/Phe + Phe/Phe	367 (46.6)	468 (57.4)	0.63 (0.52-0.77)	5.5×10^-6^
Val57Ile					
Guangxi population		n = 348	n = 359		
	Val/Val	267 (76.7)	259 (72.1)	1.00 (Reference)	
	Val/Ile	75 (21.6)	91 (25.4)	0.79 (0.55-1.13)	0.20
	Ile/Ile	6 (1.7)	9 (2.5)	0.75 (0.26-2.19)	0.63
	Val/Ile + Ile/Ile	81 (23.3)	100 (27.9)	0.79 (0.56-1.12)	0.18

OR, odds ratio. CI, confidence interval. ORs and 95% CIs were calculated by logistic regression analyses with adjustment for age, sex, smoking and drinking status, pack-years of smoking, family history of HCC and population, where appropriate.

We then went forward to genotype Ile31Phe and validate the finding, i.e., decreased susceptibility of Ile/Phe + Phe/Phe genotype to HCC, in a further sample set, the Guangdong population (Table 1). Overall, the genotypic frequencies of Ile31Phe observed in the Guangdong population were very comparable to those from the Guangxi population, and conformed to the HWE (*P* = 0.097and 0.36, for cases and controls, respectively). Again, there was an excess of Ile/Phe + Phe/Phe genotype in controls than in cases (57.7% vs. 46.8%; OR, 0.64; 95% CI, 0.49-0.83; *P* = 8.0 × 10^-4^). When the Guangxi and Guangdong populations were combined, the pooled OR for Ile/Phe + Phe/Phe genotype was 0.63 (95% CI, 0.52-0.77; *P* = 5.5×10^-6^), compared with Ile/Ile genotype (Table 1).

### Stratification analyses of association between Ile31Phe and HBV-related HCC

We further investigated the effect of *AURKA* Ile31Phe on HBV-related HCC with stratification by age, sex, status of smoking and drinking, pack-years of smoking, and family history (Supplementary Table 3). Although the susceptibility to HBV-related HCC associated with Ile/Ile genotype was more pronounced in females in the Guangdong population (*P*heterogeneity = 0.038), this was not the case in the Guangxi population (*P*heterogeneity = 0.13). The susceptibility to HBV-related HCC associated with Ile/Ile genotype appeared to be more pronounced in subjects who were older (>45 years), negative for first-degree family history of HCC, and heavier smokers (>21 pack-years); however, the difference could be attributed to chance, with all *P* values for homogeneity greater than 0.05. In accordance with the results that no appreciable variation of the effect across the subgroups stratified by age, case-only analyses did not show any differences in mean ages at diagnosis (± SD, years) in the 3 genotype groups (in the Guangxi population, 45.9 ± 11.4 years for Ile/Ile, 47.2 ± 12.2 years for Ile/Phe, and 45.0 ± 11.1 years for Phe/Phe, *P* = 0.39; in the Guangdong population, 45.9 ± 11.4 years for Ile/Ile, 47.2 ± 12.2 years for Ile/Phe, and 45.0 ± 11.1 years for Phe/Phe, *P* = 0.39). The susceptibility to HBV-related HCC associated with Ile/Ile genotype was more pronounced in smokers and drinkers (*P*heterogeneity = 8.4 × 10^-3^ and 1.8 × 10^-4^, for smoking status and drinking status, respectively).

## DISCUSSION

In this study, we for the first time reported the genetic association between the *AURKA* Ile31Phe and HBV-related HCC risk among Chinese, confirming the initial hypothesis that the *AURKA* gene may play a role in the pathogenesis of this malignancy. Furthermore, we found that the susceptibility to HBV-related HCC associated with Ile/Ile genotype was more pronounced in smokers and drinkers. One hypothesis to explain the interactions is that occurrence of larger numbers of transformed cells caused by drinking or smoking in liver probably increase the possibility that one of these cells will become malignant under the condition of higher potential of cell growth and transformation due to the Ile/Ile genotype of Ile31Phe.

Previously, a case-control study on the association between *AURKA* Ile31Phe and susceptibility to HCC has been performed by Akkiz *et al*. in a Turkish population [46]. The main finding in this study is that the proportion of Ile/Phe and Ile/Ile genotypes of Ile31Phe in patients with HCC was significantly higher than that in subjects without HCC (39.8% vs. 22.7%; *P* = 0.0030; OR =2.26, 95% CI, 1.31-3.90) [46]. However, due to study size and the heterogeneity of the cohort (e.g., 77 cases were HBsAg positive and 30 cases were anti-HCVAb positive), the association in the study of Akkiz *et al*. should have performed an independent replication. In addition, failure to match the case and control groups in that study for important confounding factors, such as virus infection, may lead to spurious results because of population stratification.

Since prevalence of HCC has distinct geographical distributions, it would be interesting to know the frequency of disease associated variant in other populations. With genotypes derived from the present and previous studies and the HapMap populations, one can find that the at-risk genotype Ile/Ile of *AURKA* Ile31Phe is more frequent among individuals of Chinese descent (40.0%) and Japanese descent (34.1%) relative to those of European descent (3.3%) and African descent (3.3%). It remains to be determined whether these differences between ethnic groups influence susceptibility to HCC.

Although the exact mechanism by which *AURKA* Ile31Phe influences the susceptibility to HBV-related HCC requires further investigation, the genetic association between Ile31Phe and the onset of HCC is biologically plausible. AURKA is a member of the Aurora/Ipl1p family of mitotically regulated serine/threonine kinases that are key regulators of chromosome segregation and cytokinesis [47]. Several studies have demonstrated that both mRNA and protein levels of AURKA were elevated in tumorous tissues of patients with HCC when compared with paired adjacent nontumorous liver tissues [28, 29]. Functional studies have revealed that the downregulation of AURKA expression in HCC cells can inhibit *in vitro* cellular proliferation and *in vivo* tumorigenicity. In mouse liver cancer, the expression of AURKA can be suppressed at transcriptional level by p53 and at post-transcriptional level by SIRT2. Furthermore, AURKA can interact physically with MYC to mediate its stabilization in TP53-altered hepatocytes in the context of chronic liver damage in mice, which would overcome G_2_/M cell-cycle arrest and promote tumor cell survival [27]. In addition, knockdown of AURKA induces G_2_/M arrest of HepG2 cells by upregulating FoxO1 in a p53-dependent manner [48], and leads to cell death inHepG2 cells probably via activation of apoptotic pathways in a caspase-dependent manner [49].

Given the role of AURKA in the development of HCC, together with the earlier described functional relevance of Ile31Phe in modulation of transforming property, we expect that individuals who carry the protective 31Phe allele, and thus might change AURKA function through modifying interactions with its binding partners and/or downstream apoptotic pathways, may be at lower susceptibility to developing HCC. If carrying the Ile/Ile homozygous genotype is regarded as a risk factor for the development of HCC, then by Adams's formula [50], the population attributable fraction (PAF) can be estimated. The PAF calculated using the relative risk (OR, 1.59; 95% CI, 1.30-1.92) and the Ile/Ile genotype frequency in the pooled controls (42.6%; Table 1) indicates that 20.1% (95% CI, 11.3-28.2%) of elevation in risk of HCC can be attributed to the susceptible effect of the Ile/Ile genotype. Based on the calculated PAF, the *AURKA* Ile31Phe polymorphism is unlikely to be appropriate for risk prediction testing. However, with more susceptibility loci identified, and interaction effects among such loci together with other HCC risk factors taken into account, the prediction of HCC occurrence may become more accurate and clinically usable.

Previous studies have reported several genetic polymorphisms associated with the progression of HCC patients [51, 52]. Thus, we investigated whether the *AURKA* polymorphisms was associated with the prognosis of patients with HBV-related HCC. A case-only data set was obtained from the GEO database (www.ncbi.nlm.nih.gov/geo/, accession number GSE38323), which containing 215 patients with HBV-related HCC from Korea for whom death or survival information was available. Using multivariate analyses, we found that the overall survival time was similar among patients with different genotypes of Ile31Phe (Ile/Ile vs. Ile/Phe + Phe/Phe, *P* = 0.10, hazard ratio (HR) = 1.44, 95% CI = 0.95-2.20; Supplementary Figure 1 and data not shown). On the contrary, as compared with Val/Ile + Ile/Ile genotypes of Val57Ile, Val/Val was significantly associated with shorter overall survival time of patients with HBV-related HCC (*P* = 0.041, HR = 1.80, 95% CI = 1.02-3.16). However, these preliminary findings may be biased due to the limited number of patients with HBV-related HCC in this sample set, and warrant confirmation in future studies.

Recently, conformation-changing AURKA inhibitors, with one of them currently being tested in early clinical trials, have been reported to suppress tumor growth and prolong survival in mice bearing xenograft tumors of human HCC cells [27]. Therefore, the functional polymorphisms of the *AURKA* gene need to be considered as a possible modification factor for cancer treatments using such inhibitors and for subsequent clinical trials.

This study has two potential limitations. First, the patients with HCC were recruited from the hospital, while the controls were selected from the community population. Therefore, inherent selection bias might exist. Second, although the highly significant association between *AURKA* Ile31Phe and susceptibility to HCC was derived from a biologically based a priori hypothesis, our initial findings require independent verification in populations with large number of subjects and of different ancestry, with appropriate study design.

In conclusion, our results reveal an association for the first time between the functional polymorphism Ile31Phe in the *AURKA* gene and susceptibility to HBV-related HCC, and, the genotypes containing 31Phe allele, which has less potential for malignant transformation than the 31Ile allele, seem to be a genetic protective factor for the HBV-related HCC risk among Chinese. If confirmed by other studies, knowledge of genetic factors contributing to the pathogenesis of HCC as presented here may have implications for the cancer screening and treatment of this malignance in the future.

## MATERIALS AND METHODS

### Study subjects

This study included two independent case-control populations (Supplementary Table 1). All the cases and controls were unrelated ethnic adult Chinese, and residents in Guangxi province (Guangxi population) and Guangdong province (Guangdong population), respectively. The diagnosis of HCC, the inclusion and exclusion criteria for the cases and controls were described in detail previously [43–45]. Briefly, both the HCC cases and controls were chronic HBV carriers positive for both hepatitis B surface antigen and antibody immunoglobulin G to hepatitis B core antigen for at least 6 months. For Guangdong population, 751 patients with HCC and 509 control subjects were consecutively recruited from August 2003 to November 2007 at the Sun Yat-sen University Cancer Center (Guangzhou, China) at Guangdong province located in the southern China. All the cases and controls were self-reported Cantonese residing at the Guangzhou city and its surrounding regions. For Guangxi population, a total of 348 patients with HBV-related HCC were enrolled between July 2002 and January 2008 at the Guangxi Cancer Hospital (Nanning, China) [43]. All cases were residents in Fusui county and the surrounding regions at Guangxi province, a well-known high-risk region for HCC located in southern China. The 359 controls were randomly selected from a community cancer screening program for early detection of cancer conducted in the same regions during the same time period as the HCC cases were enrolled.

At recruitment, informed consent was obtained from each subject. This study was performed with the approval of the Ethical Committee of Beijing Institute of Radiation Medicine (Beijing, China).

### Genotyping

In Guangxi population, the Ile31Phe was genotyped by PCR-restriction fragment length polymorphism (RFLP), as described previously [34]. An amplification of a 165-bp fragment was performed using primers as listed in Supplementary Table 4. The PCR product was digested overnight with 2U of *Apo*I enzyme (New England BioLabs, Beverly, MA) at 50°C, and then separated on 3% agarose gel. The sequence of six bases beginning from the 153rd bp was a natural *Apo*I restriction site and the Ile allele located at the 89th bp created a new *Apo*I restriction site. Thus, homozygotes carrying Phe/Phe genotype appeared as two bands (153 and 12bp), but only a 153-bp band could be visualized in the agarose gel. Homozygotes carrying Ile/Ile genotype appeared as three bands (89, 64 and 12 bp), and only 89-bp and 64-bp bands could be seen. Heterozygotes carrying Ile/Phe genotype had four bands (153, 89, 64 and 12 bp), and the first three bands could appear.

The Ile31Phe and Val57Ile were genotyped in Guangdong population and Guangxi population respectively, by Sequenom MassARRAY system according to the manufacturer's instructions [53]. The MassARRAY system is based on a single-base primer extension technology. This platform employs the matrix-assisted laser desorption ionization time-of-flight (MALDI-TOF) mass spectrometry to measure the mass of the extension products and then correlates the detected mass with a specific genotype. Primers used for polymerase chain reaction (PCR) and single-base extension were designed by the Assay Designer software (Sequenom) (Supplementary Table 4). Genotype data were automatically called and visualized for clustering by Typer 4.0 (Sequenom).

In both populations, genotyping was performed without knowledge of case or control status. Ile31Phe and Val57Ile had genotyping call rates of 100%. A masked, random sample of 15% of the cases and controls from both Guangxi and Guangdong populations was tested by PCR direct sequencing, and all results had 100% concordance.

### Statistical analysis

Hardy-Weinberg equilibrium (HWE) was analyzed using the web-based tool SNPstats (http://bioinfo.iconcologia.net/SNPstats). Differences in the distributions of demographical characteristics between the cases and the controls were evaluated using the Student's *t*-test (for continuous variables) and χ^2^ test (for categorical variables).

The association between 31Ile and the risk of HCC was estimated by computing the odds ratio (OR) and 95% confidence interval (95% CI) using binary logistic regression analyses with adjustment for age, sex, smoking and drinking status, pack-years of smoking and family history of HCC in case-control populations, where appropriate. The potential modification effects of Ile31Phe on HCC risk were assessed for age, sex, smoking and drinking status, pack-years of smoking and family history of HCC by the addition of interaction terms in the logistic regression model and by separate analyses of subgroups of subjects stratified by these factors.

All *P* values were two-sided, and a level of *P* < 0.05 was considered statistically significant. These analyses were performed using the SPSS software (version 10.0; SPSS Inc, Chicago, IL).

This work was supported by grants from the Major Project of Chinese National Programs for Fundamental Research and Development (No. 2013CB910301), National Natural Science Foundation of China (No. 91440206, 81125017, 81222027 and 81472274), Program of Beijing Municipal Commission for Science and Technology for Frontier Technology of Life Science (No. Z141100000214014), Chinese Key Project for Infectious Diseases (No. 2017ZX10302202 and 2017ZX10203205), Program of International S&T Cooperation (No. 2014DFB30020) and Beijing Science & Technology NOVA Program (No. 2010B006).

## SUPPLEMENTARY MATERIALS FIGURES AND TABLES






