# Supplementary material for: Association between the functional polymorphism Ile31Phe in the AURKA gene and susceptibility of hepatocellular carcinoma in chronic hepatitis B virus carriers
Source: Oncotarget. 2017 Jun 27;8(33):54904–12. doi: 10.18632/oncotarget.18613 (PMC5589629; doi:10.18632/oncotarget.18613)
Supplement: Supplementary file 1 [file oncotarget-08-54904-s001.pdf]

Association between the functional polymorphism Ile31Phe in the *AURKA* gene and susceptibility of hepatocellular carcinoma in chronic hepatitis B virus carriers

SUPPLEMENTARY MATERIALS

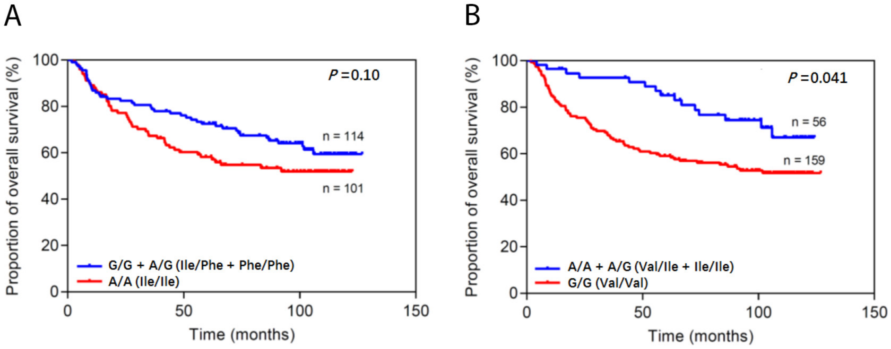

Supplementary Figure 1: Kaplan-Meier estimates of survival time for the individuals with HCC stratified by genotypes of (a) Ile31Phe and (b) Val57Ile based on GSE38323 data. This dataset of GSE38323 contains 215 patients with HBV-related from Korea. The genotypes of Ile31Phe were unavailable in this dataset, thus we used rs6127737 as a proxy SNP, which showed strong linkage disequilibrium with Ile31Phe (r^2 = 0.94 in Asian population based on 1000 Genomes Project data). Hazard ratios (HRs) and their 95% confidence intervals (CIs) were calculated using Cox proportional hazard models, and the Kaplan-Meier survival estimates were plotted. In multivariate analyses, we considered genotypes, age at diagnosis, gender and TNM stage.

Supplementary Table 1: Summary description of the samples used in this study.
Supplementary File 1

Supplementary Table 3: Stratification analyses of association between Ile31Phe and HCC risk in two case-control populations.
Supplementary File 1

Supplementary Table 2: Association of estimated haplotypes in the *AURKA* gene with hepatocellular carcinoma in the Guangxi case-control population.

Haplotype	Cases, 2n (%) (n = 348)	Controls, 2n (%) (n = 359)	OR (95% CI)	*P* value [a]
Ile-Val	505 (72.6)	472 (65.7)	1	
Phe-Val	104 (14.9)	137 (19.1)	0.69 (0.51 - 0.93)	0.016
Phe-Ile	87 (12.5)	109 (15.2)	0.77 (0.56 - 1.05)	0.10

The haplotype is in the order of Ile31Phe and Val57Ile.
[a]No correction was made for testing multiple alleles.

Supplementary Table 4: Primers and probes used for SNPs genotyping.

SNPs	Primers and probes	Sequences (5′→3′)
**By PCR-RFLP genotyping**		
Ile31Phe	Forward	CTTTCATGAATGCCAGAAAGTT
	Reverse	CTGGGAAGAATTTGAAGGACA
**By Sequenom genotyping**		
Ile31Phe	Forward	ACGTTGGATGAGGTCCAAAACGTGTTCTCG
	Reverse	ACGTTGGATGTGAGCCTGGCCACTATTTAC
	Extend	TCTCGTGACTCAGCAAT
Val57Ile	Forward	ACGTTGGATGTGAACCGGCTTGTGACTGGA
	Reverse	ACGTTGGATGTAAATAGTGGCCAGGCTCAG
	Extend	TGTGCTTGCAAAGGAA

SNP, single nucleotide polymorphism. PCR-RFLP, polymerase chain reaction- restriction fragment length polymorphism. PCR was performed with an initial 2 min at 50°C and 10 min at 95°C, followed by 40 cycles of 15 sec at 95°C and 1 min at 60°C.
