# Supplementary material for: Association between the functional polymorphism Ile31Phe in the AURKA gene and susceptibility of hepatocellular carcinoma in chronic hepatitis B virus carriers
Source: Oncotarget. 2017 Jun 27;8(33):54904–12. doi: 10.18632/oncotarget.18613 (PMC5589629; doi:10.18632/oncotarget.18613)
Supplement: Supplementary file 2 [file oncotarget-08-54904-s002.docx]

**Supplementary Table 1:** Summary description of the samples used in this study.

|  | Guangxi population | | | Guangdong population | | | |
| --- | --- | --- | --- | --- | --- | --- | --- |
|  | Cases  (n = 348) | Controls  (n = 359) | *P* | Cases  (n = 440) | Controls  (n = 456) | *P* |  |
| Age, years |  |  |  |  |  |  |  |
| Mean (SD) | 45.8 (10.6) | 41.6 (12.1) | 1.2 × 10^-6^ | 49.1 (11.5) | 47.8 (11.2) | 0.082 |  |
| ≤ 45, n (%) | 187 (53.7) | 237 (66.0) | 9.6 × 10^-4^ | 179 (40.7) | 188 (41.2) | 0.89 |  |
| > 45, n (%) | 161 (46.3) | 122 (34.0) |  | 261 (59.3) | 268 (58.8) |  |  |
| Sex, n (%) |  |  |  |  |  |  |  |
| Female | 45 (12.9) | 49 (13.6) | 0.83 | 62 (14.1) | 78 (17.1) | 0.23 |  |
| Male | 303 (87.1) | 310 (86.4) |  | 378 (85.9) | 378 (82.9) |  |  |
| Smoking status, n (%) |  |  |  |  |  |  |  |
| Non-smoker | 224 (64.4) | 208 (57.9) | 0.090 | 44 (10.0) | 97 (21.3) | 0.37 |  |
| Smoker | 124 (35.6) | 151 (42.1) |  | 47 (10.7) | 81 (17.8) |  |  |
| Unknown | 0 | 0 |  | 349 (79.3) | 278 (61.0) |  |  |
| Smoking level, pack-years |  |  |  |  |  |  |  |
| Mean (SD) | 20.5 (19.7) | 20.3 (19.0) | 0.93 | 19.8 (16.3) | 22.3 (13.2) | 0.35 |  |
| ≤ 21, n (%) | 88 (71.0) | 109 (72.2) | 0.89 | 36 (76.6) | 47 (58.0) | 0.037 |  |
| > 21, n (%) | 36 (29.0) | 42 (27.8) |  | 11 (23.4) | 34 (42.0) |  |  |
| Unknown | 0 | 0 |  | 0 | 0 |  |  |
| Drinking status, n (%) |  |  |  |  |  |  |  |
| Non-drinker | 256 (73.6) | 262 (73.0) | 0.87 | 48 (10.9) | 100 (21.9) | 0.61 |  |
| Drinker | 92 (26.4) | 97 (27.0) |  | 43 (9.8) | 78 (17.1) |  |  |
| Unknown | 0 | 0 |  | 349 (79.3) | 278 (61.0) |  |  |
| First-degree family history of HCC, n (%) | | |  |  |  |  |  |
| Negative | 292 (83.9) | 338 (94.2) | 1.2 × 10^-5^ | 78 (17.7) | 158 (34.6) | 0.56 |  |
| Positive | 56 (16.1) | 21 (5.8) |  | 13 (3.0) | 20 (4.4) |  |  |
| Unknown | 0 | 0 |  | 349 (79.3) | 278 (61.0) |  |  |

SD, standard deviation. *P* values were calculated by *t* test (2-sided) for means of age and smoking level, and *χ*^2^ test for other variables. Age-stratified analyses were based on the mean age in overall controls (≤ 45 or > 45 years). Pack-years-stratified analyses were based on the mean pack-years in overall controls (≤ 21 or > 21 pack-years).
