# Supplementary material for: Association between the functional polymorphism Ile31Phe in the AURKA gene and susceptibility of hepatocellular carcinoma in chronic hepatitis B virus carriers
Source: Oncotarget. 2017 Jun 27;8(33):54904–12. doi: 10.18632/oncotarget.18613 (PMC5589629; doi:10.18632/oncotarget.18613)
Supplement: Supplementary file 3 [file oncotarget-08-54904-s003.docx]

**Supplementary Table 3**: Stratification analyses of association between Ile31Phe and HCC risk in two case-control populations.

| Category | Guangxi, n (%) | Guangdong, n (%) |
| --- | --- | --- |
|  | Case / Control | Case / Control |
| **Sex** |  |  |
| Male | n = 303 / n = 310 | n = 378 / n = 378 |
| Ile/Ile | 161 (53.1) / 134 (43.2) | 194 (51.3) / 164 (43.4) |
| Ile/Phe+Phe/Phe | 142 (46.9) / 176 (56.8) | 184 (48.7) / 214 (56.6) |
| OR (95% CI) | 0.69 (0.49-0.97) | 0.73 (0.54-0.97) |
| *P* value | 0.030 | 0.028 |
| Female | n = 45 / n = 49 | n = 62 / n = 78 |
| Ile/Ile | 26 (57.8) / 20 (40.8) | 40 (64.5) / 29 (37.2) |
| Ile/Phe+Phe/Phe | 19 (42.2) / 29 (59.2) | 22 (35.5) / 49 (62.8) |
| OR (95% CI) | 0.47 (0.20-1.13) | 0.31 (0.15-0.64) |
| *P* value | 0.087 | 0.0011 |
| *P*_heterogeneity_ | 0.13 | 0.038 |
| **Age** |  |  |
| ≤45 years | n = 187 / n = 240 | n = 179 / n = 188 |
| Ile/Ile | 104 (55.6) / 116 (48.3) | 94 (52.5) / 84 (44.7) |
| Ile/Phe+Phe/Phe | 83 (44.4) / 124 (51.7) | 85 (47.5) / 104 (55.3) |
| OR (95% CI) | 0.80 (0.53-1.21) | 0.75 (0.49-1.14) |
| *P* value | 0.29 | 0.18 |
| >45 years | n = 161 / n = 119 | n = 261 / n = 268 |
| Ile/Ile | 83 (51.5) / 38 (31.9) | 140 (53.6) / 109 (40.7) |
| Ile/Phe+Phe/Phe | 78 (48.5) / 81 (68.1) | 121 (46.4) / 159 (59.3) |
| OR (95% CI) | 0.36 (0.21-0.62) | 0.58 (0.41-0.83) |
| *P* value | 1.0 × 10^-4^ | 0.0024 |
| *P*_heterogeneity_ | 0.051 | 0.36 |
| **Smoking status** |  |  |
| Smokers | n = 124 / n = 151 |  |
| Ile/Ile | 72 (58.1) / 52 (34.4) |  |
| Ile/Phe+Phe/Phe | 52 (41.9) / 99 (65.6) |  |
| OR (95% CI) | 0.38 (0. 22-0.63) |  |
| *P* value | 2.0× 10^-4^ |  |
| Nonsmokers | n = 224 / n = 208 |  |
| Ile/Ile | 115 (51.3) / 102 (49.0) |  |
| Ile/Phe+Phe/Phe | 109 (48.7) / 106 (51.0) |  |
| OR (95% CI) | 0.88 (0. 59-1.31) |  |
| *P* value | 0.53 |  |
| *P*_heterogeneity_ | 8.4× 10^-3^ |  |
| **Smoking level (pack-years)** |  |  |
| ≤ 21 | n = 88 / n = 109 |  |
| Ile/Ile | 50 (56.8) / 40 (36.7) |  |
| Ile/Phe+Phe/Phe | 38 (43.2) / 69 (63.3) |  |
| OR (95% CI) | 0.47 (0.25-0.87) |  |
| *P* value | 0.015 |  |
| >21 | n = 36 / n = 42 |  |
| Ile/Ile | 22 (61.1) / 12 (28.6) |  |
| Ile/Phe+Phe/Phe | 14 (38.9) / 30 (71.4) |  |
| OR (95% CI) | 0.21 (0.07-0.58) |  |
| *P* value | 2.0× 10^-3^ |  |
| *P*_heterogeneity_ | 0.29 |  |
| **Drinking status** |  |  |
| Drinkers | n = 92 / n = 97 |  |
| Ile/Ile | 63 (68.5) / 33 (34.0) |  |
| Ile/Phe+Phe/Phe | 29 (31.5) / 64 (66.0) |  |
| OR (95% CI) | 0.25 (0. 13-0.47) |  |
| *P* value | 1.4 × 10^-5^ |  |
| Nondrinkers | n = 256 / n = 262 |  |
| Ile/Ile | 124 (48.4 / 121 (46.2) |  |
| Ile/Phe+Phe/Phe | 132 (51.6) / 141 (53.8) |  |
| OR (95% CI) | 0.93 (0. 64-1.34) |  |
| *P* value | 0.69 |  |
| *P*_heterogeneity_ | 1.8 × 10^-4^ |  |
| **First-degree family history of HCC, n (%)** | |  |
| Negative | n = 292 / n = 338 |  |
| Ile/Ile | 157 (53.8) / 142 (42.0) |  |
| Ile/Phe+Phe/Phe | 135 (46.2) / 196 (58.0) |  |
| OR (95% CI) | 0.60 (0. 43-0.83) |  |
| *P* value | 2.0 × 10^-3^ |  |
| Positive | n = 56 / n = 21 |  |
| Ile/Ile | 30 (53.6) / 12 (57.1) |  |
| Ile/Phe+Phe/Phe | 26 (46.4) / 9 (42.9) |  |
| OR (95% CI) | 1.23 (0. 39-3.95) |  |
| *P* value | 0.72 |  |
| *P*_heterogeneity_ | 0.22 |  |

OR, odds ratio. CI, confidence interval. *P* values, ORs and 95% CIs were calculated by logistic regression with the Ile/Ile genotype as the reference group and adjusted for age, sex, smoking and drinking status, pack-years of smoking, and family history of HCC, where appropriate. Age-stratified analyses were based on the mean age in overall controls (≤ 45 or > 45 years). Pack-years-stratified analyses were based on the mean pack-years in overall controls (≤ 21 or > 21 pack-years). Because majority of the Guangdong subjects lack the information about smoking and drinking status, pack-years of smoking, and family history of HCC, stratified association analyses were not performed within these strata.
